# ^131^I-Traced PLGA-Lipid Nanoparticles as Drug Delivery Carriers for the Targeted Chemotherapeutic Treatment of Melanoma

**DOI:** 10.1186/s11671-017-2140-7

**Published:** 2017-05-19

**Authors:** Haiyan Wang, Weizhong Sheng

**Affiliations:** 1 Department of Nuclear Medicine, Shanghai East Hospital, Tongji University School of Medicine, Shanghai, 200120 China; 2 Department of General Surgery, Zhongshan Hospital, Fudan University, Shanghai, 200032 China

**Keywords:** Nanocarrier, PLGA-lipid, ^131^I, Biodistribution, Drug delivery, Melanoma

## Abstract

Herein, folic acid (FA) conjugated Poly(d,l-lactide-co-glycolide) (PLGA)-lipid composites (FA-PL) were developed as nanocarriers for the targeted delivery of insoluble anti-cancer drug paclitaxel (PTX), resulting FA-PLP nanoparticles. Furthermore, ^131^I, as a radioactive tracer, was used to label FA-PLP nanoparticles (FA-PLP-^131^I) to evaluate their cell uptake activity, in vivo blood circulation, and biodistribution. The FA-PLP-^131^I nanoparticles had a spherical morphology with great stability, a narrow size distribution (165.6 and 181.2 nm), and −22.1 mV in average zeta potential. Confocal laser scanning microscopy indicated that the targeting molecule FA promotes PLP-^131^I uptake by melanoma B16F10 cells, which was further confirmed by the cell incorporation rate via ^131^I activity detection as measured by a gamma counter. FA-PLP-^131^I without PTX (FA-PL-^131^I) shows minor cytotoxicity, good biocompatibility, while FA-PLP-^131^I was demonstrated to have efficient cell viability suppression compared to free PTX and PLP-^131^I. Following intravenous injection, the blood circulation half-life of free PTX (*t*_1/2_ = 5.4 ± 0.23 h) was prolonged to 18.5 ± 0.5 h by FA-PLP-^131^I. Through FA targeting, the tumor uptake of FA-PLP-^131^I was approximately 4.41- and 12.8-fold higher compared to that of PLP-^131^I and free PTX-^131^I, respectively. Moreover, following 40 days of treatment, FA-PLP-^131^I showed an improved tumor inhibition effect compared to free PTX and PLP-^131^I, with no relapse and no remarkable systemic in vivo toxicity. The results demonstrate that the ^131^I-labeled PLGA-lipid nanoparticle can be simultaneously applied for targeted drug delivery and reliable tracking of drugs in vivo.

**Electronic supplementary material:**

The online version of this article (doi:10.1186/s11671-017-2140-7) contains supplementary material, which is available to authorized users.

## Background

One of the most aggressive skin cancers, melanoma, originates from the malignant transformation of melanocytes [1, 2]. Given its easy relapse and high potential for metastasis, the 5-year survival of metastasized melanoma patients is only 10%. To date, the most common treatment for melanoma patients is chemotherapy, which is accompanied by undesirable severe side effects, low bioavailability, poor tumor selectivity, and dose-limiting systemic toxicity, presenting major challenges in tumor chemotherapy [3, 4].

Paclitaxel (PTX), a natural plant extract derived from the dried roots, branches, leaves, and bark of taxus, a genus of coniferous trees [5, 6], shows effective antitumor activity towards several kinds of tumors, including ovarian carcinoma and lung cancer [7–9]. Additionally, PTX has also been reported as efficient against human melanoma [10, 11]. Nevertheless, in addition to the abovementioned drawbacks of chemotherapeutic drugs, a Cremophor EL and dehydrated alcohol (1:1, *v*/*v*) mixture is used in current clinical practice as the dilution medium for PTX, which may lead to serious side effects, including hypersensitivity [12, 13]. Therefore, the development of novel strategies to enhance the aqueous solubility and tumor accumulation of chemotherapeutic agents to reduce their peripheral exposure and minimize their in vivo toxicity is paramount. The recent development of biocompatible drug nanocarriers provides the potential to enhance the physiological stability of PTX [14–16]. Additionally, the conjugation of target molecules to these nanocarriers would allow the selective delivery of chemotherapeutic agents to tumor sites with reduced peripheral exposure through a tumor cell surface receptor-mediated targeted effect [17–19].

Herein, we prepared a Poly(d,l-lactide-co-glycolide) (PLGA)-lipid composite that covalently conjugates folic acid (FA: a tumor targeting molecule) and encapsulates PTX as a chemotherapeutic drug for melanoma treatment. Moreover, ^131^I, a radioactive marker, was used to radiolabel the PLGA-lipid nanoparticles to clearly assess their in vivo behavior. Because of its negative beta emission, physical half-life, and wide range of decay properties, ^131^I is commonly used as a radiolabel in clinic [20–22]. The morphology, stability, and dispersity of the ^131^I-labeled PLGA-lipid nanoparticles (FA-PLP-^131^I) were evaluated in vitro. Furthermore, the cell uptake, blood circulation, and biodistribution of FA-PLP-^131^I were investigated by measuring the radioactivity of ^131^I. Moreover, the targeted anticancer efficacy of FA-PLP-^131^I was studied in vitro and in vivo. The results indicate that FA-PLP-^131^I may be a versatile nanoplatform for use as a potential tumor drug delivery nanocarrier for chemotherapeutic drugs.

## Methods

### Materials

Poly(d,l-lactide-co-glycolide) (PLGA, MW: 5000–15,000, lactide:glycolide (50:50)) and chloramine-T were purchased from Sigma Aldrich (St. Louis, MO, USA). Na^131^I was obtained from Atomic Hitech (Beijing, China). PTX (99%) and 4′,6-diamidino-2-phenylindole (DAPI) were purchased from Aladdin Chemical Reagent Co., Ltd. (Shanghai, China). Soybean lecithin consisting of 90–95% phosphatidylcholine, 1,2-distearoyl-sn-glycerol-3-phosphoethanolamine-*N*-[folate (polyethylene glycol)-2000] (DSPE-PEG_2000_-FA), and 1,2-distearoyl-sn-glycero-3-phosphoethanolamine-*N*-[carboxy (polyethylene glycol)-2000] (DSPE-PEG_2000_-COOH) were obtained from Avanti (Alabaster, AL, USA). All cell culture reagents were purchased from Sigma Aldrich.

### Preparation of FA-PLP Nanoparticles

FA-PLP nanoparticles were synthesized by a self-assembly nanoprecipitation method [23]. In detail, 10 mg PTX was dissolved in 1 mL ethanol and 2 mg PLGA was dissolved in 1 mL dichloromethane. Following mixing them, lecithin/DSPE-PEG_2000_-FA (4:1) ethanol aqueous solution (4 wt%) was added drop-wise into the mixture solution for 4 h gentle stirring at 25 °C. The mixture was filtered and washed three times with deionized water using a Millipore ultrafiltration centrifuge tube to remove the non-encapsulated drug and organic solvent. As a control, nanoparticles without FA molecule grafting were prepared through the same method, replacing DSPE-PEG_2000_-FA with DSPE-PEG_2000_-COOH. The purified FA-PLP nanoparticles were stored at 4 °C until further use.

### Preparation of ^131^I-Labeled FA-PLP Nanoparticles

Radioactive FA-PLP (FA-PLP-^131^I) nanoparticles were prepared through the chloramine-T oxidation method [24]. A mixture of 1 mL FA-PLP (1 mg/mL), 500 μCi Na^131^I (which is the maximum radioactivity that can be grafted onto FA-PLP), and 100 μL of 5 mg/mL chloramine-T were reacted in a pH 7.5 phosphate buffer for 10 min at room temperature. The reaction was then quenched by adding 200 μL of sodium metabisulfite (5 mg/mL). ^131^I-labeled PLP and PTX were prepared following the same procedure. They were purified using a centrifuge tube (Millipore) to remove the remaining free Na^131^I until no gamma activity was detectable in the filtrate solution. The radiolabeling yield and purity of the labeled nanoparticles were analyzed using a gamma counter (LKB gamma 1261; LKB Instruments).

### Characterization

UV–vis absorption spectra were recorded using a UV–vis spectrophotometer (UV7502, Shanghai Advanced Photoelectric Technology Co., Ltd., Shanghai, China). Transmission electron microscopy (TEM) images were collected on a Zeiss LIBRA 120 TEM. The size, zeta potential, and polydispersity index (PDI) of nanoparticles were detected by dynamic light scattering (DLS) analysis using a Zetasizer Nano ZS (Malvern Instruments). Optical photographs were taken with a Nikon D3200 digital camera.

### Cell Culture

The mouse melanoma cell line B16F10 was obtained from the Cell Bank of the Type Culture Collection of the Chinese Academy of Sciences (Shanghai, China) and cultured in DMEM containing 10% fetal bovine serum and 100 U/mL of penicillin/streptomycin in a humidified 5% CO_2_ atmosphere at 37 °C.

### In Vitro Incorporation Assays

Time-dependent in vitro incorporation assays were performed to determine the optimum cell binding efficiency times for the ^131^I-labeled compounds [25]. B16F10 cells were seeded in 24-well plates at 1 × 10^5^ cells per well and cultured to confluence. Radioiodinated samples (^131^I, PL-^131^I, FA-PL-^131^I, PLP-^131^I, FA-PLP-^131^I) were prepared in DMEM media and added to the cell culture wells, separately. After 0.5, 1, 2, 4, and 6 h incubation, the cells were washed with PBS three times and their radioactivity measured with a gamma counter (Science and Technology Institute of China, Jia Branch Innovation Co., Ltd.). Incorporation values (%) were calculated as described in the previous literature [25]. Additionally, nanoparticles were labeled with the fluorescent dye fluorescein isothiocyanate (FITC, Sigma) and then incubated with B16F10 cells. The fluorescence images of cells were captured using a commercial confocal laser scanning microscope (FV1200, Olympus, Tokyo, Japan).

### In Vitro Cytotoxicity Assay

The MTT cell viability assay was used to study the cytotoxicity of PL-^131^I and FA-PL-^131^I, as well as that of free PTX, PLP-^131^I, and FA-PLP-^131^I, against B16F10 cells. Briefly, B16F10 cells were plated in 96-well plates for 24 h and exposed to PL-^131^I and FA-PL-^131^I (with 0–100 μg/mL PLGA-lipid), or free PTX, PLP-^131^I, and FA-PLP-^131^I (at various concentrations of 0–40 μg/mL) for 24 h. The experiment was performed in triplicate. All data were expressed as the mean ± SD.

### Animal Model

Three- to 5-week-old Balb/c mice were purchased from Shanghai Slack Laboratory Animal Co., Ltd. (Shanghai, China). All animal experiments were approved by the Animal Care and Use Committee of Fudan University, which complies with the National Institutes of Health Guide for the Care and Use of Laboratory Animals.

B16F10 cells (1 × 10^6^) in PBS were injected subcutaneously into the right flank of mice. The growing tumor volume was measured using a caliper, and the tumor volume was calculated using the formula: volume = (length × width^2^)/2. When the tumor volume reached approximately 80 mm^3^, the mice were randomized into the experimental groups.

### Blood Circulation and Biodistribution Study

Healthy Balb/c mice were intravenously injected with PTX-^131^I and FA-PLP-^131^I (100 μL of 10 μCi per mouse, 5 mg/kg). Blood circulation was measured by drawing approximately 10 μL of blood from the tail of mice. The radioactivity in blood was measured using a gamma counter. In order to detect the nanoparticle biodistribution, mice bearing a B16F10 tumor were injected with PTX-^131^I, FA-PL-^131^I, PLP-^131^I, and FA-PLP-^131^I at the same dose and sacrificed 24 h after injection. The major organs were weighed and collected for gamma counting.

### In Vivo Tumor Chemotherapy

Balb/c mice bearing B16F10 tumors were injected with 150 μL of saline, free PTX, PLP-^131^I, and FA-PLP-^131^I (at the same PTX concentration, 5 mg/kg). The tumor size and body weight were measured by a caliper every 4 days. Relative tumor volumes were calculated as *V*/*V*_0_ (*V*_0_ was the tumor volume when the treatment was initiated). After approximately 40 days of treatment, mice were sacrificed and the major organs collected, fixed in 4% formalin, paraffin embedded and sliced, stained with hematoxylin and eosin, and examined under a digital microscope.

## Results and Discussion

### Preparation and Characterization of ^131^I-Labeled FA-PLP Nanoparticles

**Fig. 1:**
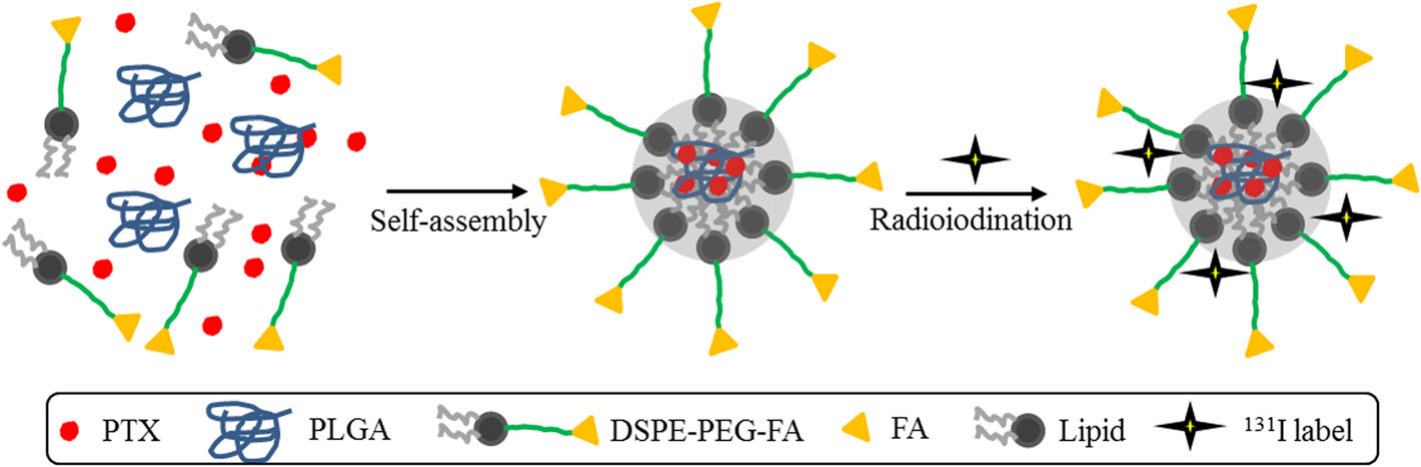
Synthesis of FA-PLP-^131^I nanoparticles

**Fig. 2:**
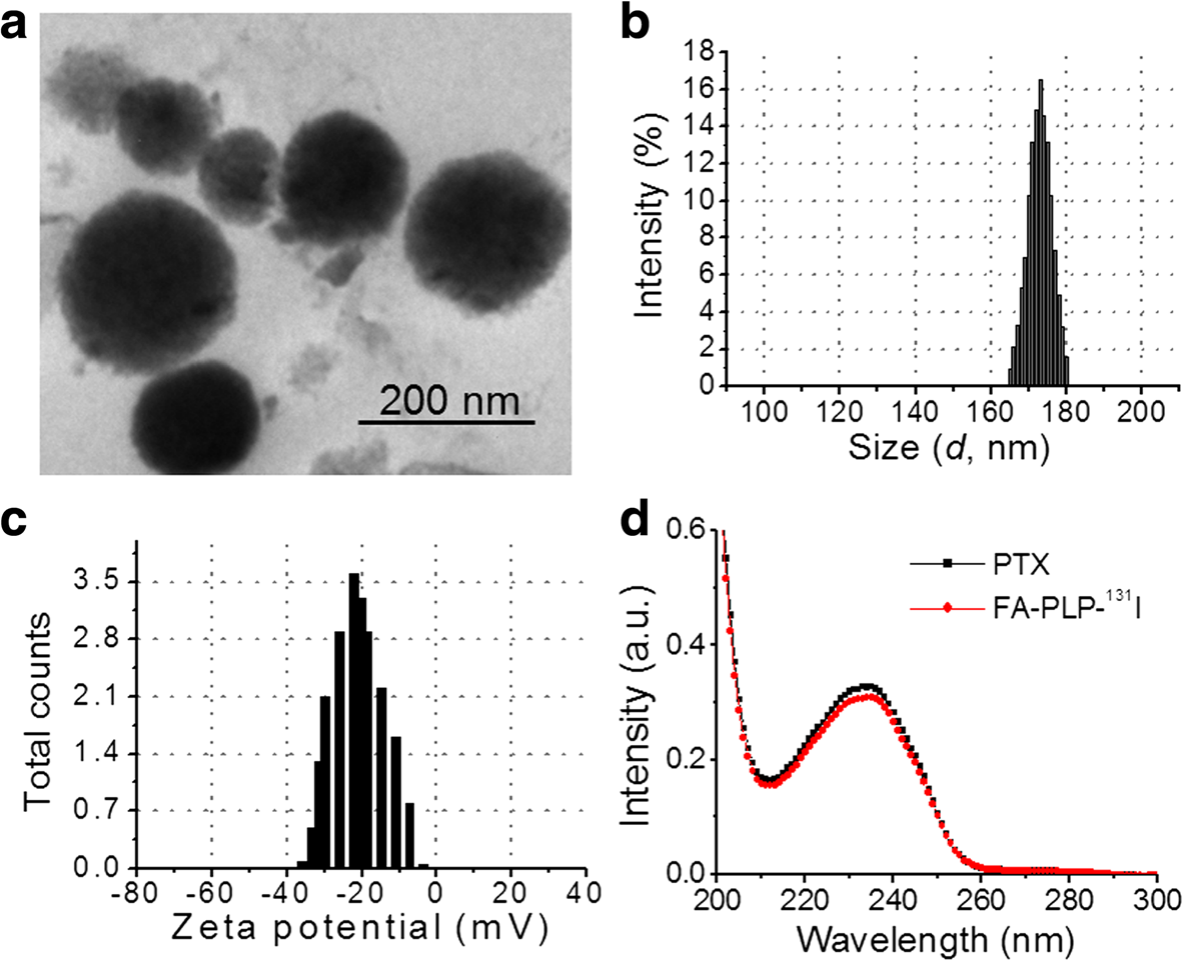
**a** TEM image of FA-PLP-^131^I. **b** Particle size and **c** zeta potential of FA-PLP-^131^I analyzed by dynamic light scattering. **d** Absorbance spectra of free PTX and FA-PLP-^131^I

The synthesis scheme of FA-PLP-^131^I nanoparticles is shown in Fig. 1. In brief, FA-PLP nanoparticles were synthesized through a self-assembly nanoprecipitation method and radioactive FA-PLP (FA-PLP-^131^I) was prepared using the chloramine-T oxidation method [23, 24]. PTX was encapsulated by the PLGA-lipid composite, which was then grafted with covalent conjugated FA on the surface of the shell via PEGylation. Finally, ^131^I was grafted onto the outer nanoparticle surface. TEM images indicated that the FA-PLP-^131^I nanoparticles had a spherical morphology of narrow size distribution (165.6 to 181.2 nm), which was confirmed by dynamic light scattering (Fig. 2a, b). The zeta potential ranged from −39.1 to −3.2 mV (Fig. 2c). The UV–vis spectra of free PTX and FA-PLP-^131^I (with same concentration of PTX) showed the same feature peak at 233 nm (Fig. 2d), indicating that PTX had been encapsulated within FA-PLP-^131^I and that encapsulation did not influence the absorbance intensity of PTX. After calculation, the encapsulation efficiency of PTX in FA-PLP-^131^I was shown to be 56.35 ± 1.6%. The radiolabeling yield of PTX-^131^I, PL-^131^I, FA-PL-^131^I, PLP-^131^I, and FA-PLP-^131^I was 45.6 ± 2.3, 52.1 ± 4.1, 48.9 ± 1.9, 56.3 ± 2.5, and 54.8 ± 2.7, respectively.

**Fig. 3:**
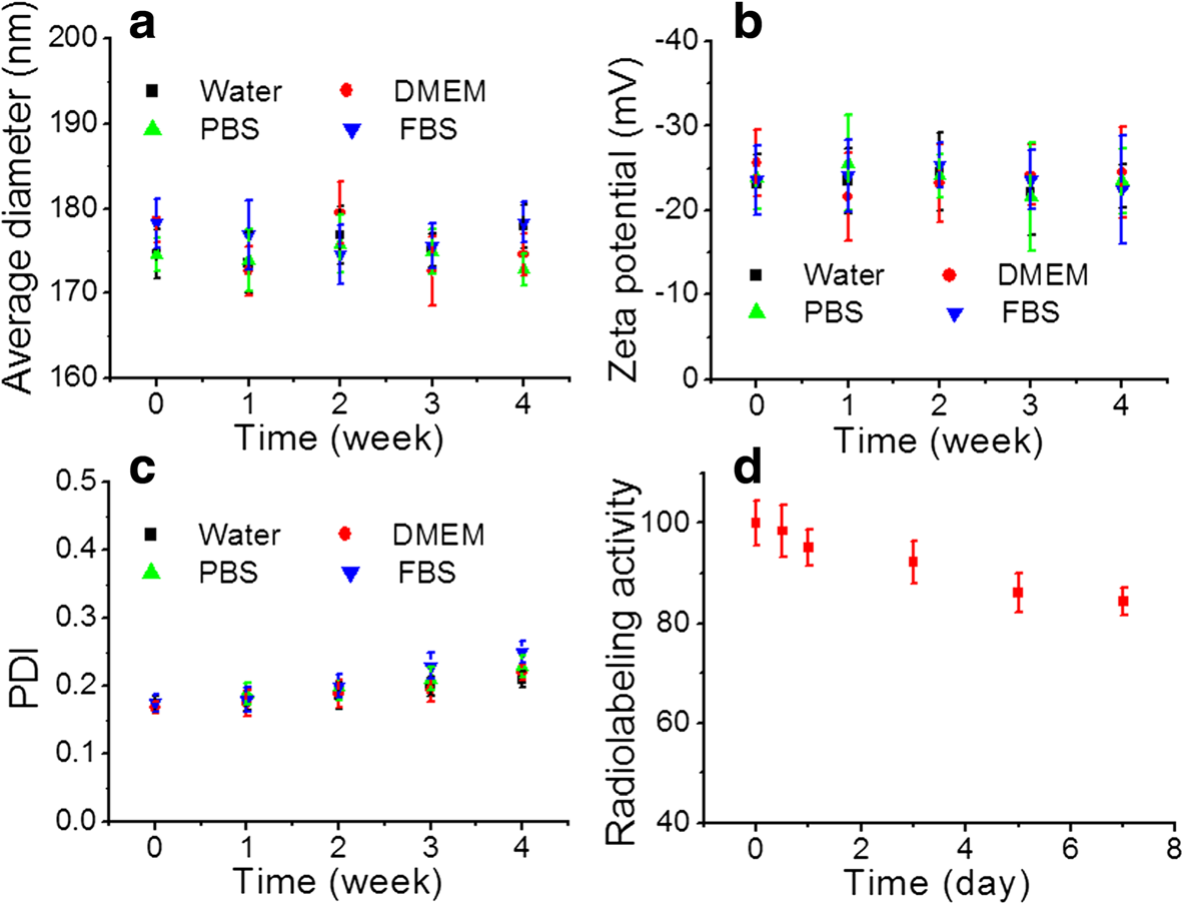
**a**, **b** Colloid stability and **c** PDI test of FA-PLP-^131^I in different media, including water, DMEM, PBS, and fetal bovine serum (FBS). **d** Radiolabeling stability curve of FA-PLP-^131^I in mouse plasma at 37 °C within 2 weeks of storage

Stability is essential to the biomedical application of nanoparticles [26]. After 4 weeks in storage, FA-PLP-^131^I dissolved in water, cell media, fetal bovine serum, and PBS displayed no changes in average size, zeta potential, PDI index (Fig. 3a–c), indicating great stability and dispersity. In addition, the radiolabeling stability of FA-PLP-^131^I was detected in mouse plasma at 37 °C (Fig. 3d), showing less than 15% de-iodination within 7 days.

### In Vitro Cellular Uptake

**Fig. 4:**
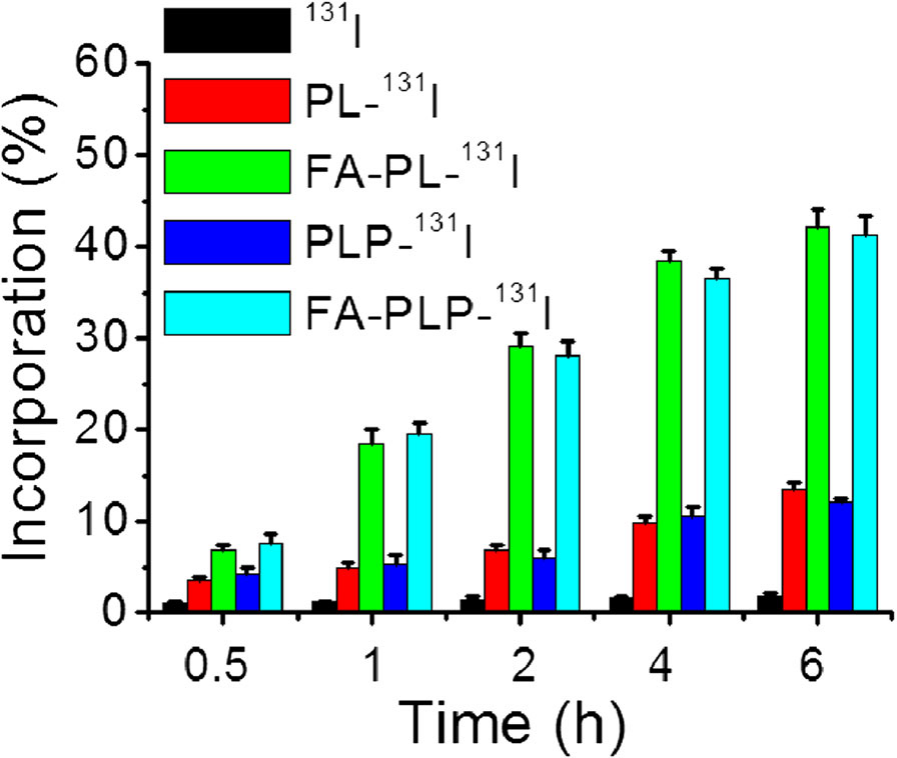
Time-dependent incorporation of ^131^I, PL-^131^I, FA-PL-^131^I, PLP-^131^I, and FA-PLP-^131^I on B16F10 cells

Figure 4 shows the in vitro time-dependent incorporation of ^131^I, PL-^131^I, FA-PL-^131^I, PLP-^131^I, and FA-PLP-^131^I in B16F10 cells measured by a gamma counter. FA-PLP-^131^I, as well as FA-PL-^131^I, showed the higher incorporation values than that of all tested time points, increasing with time. The incorporation values of FA-PLP-^131^I at 6 h were 3.12- and 23.4-fold higher than those of ^131^I and PLP-^131^I, in agreement with the results of confocal laser scanning microscopy images of B16F10 cells incubated with fluorescein isothiocyanate-labeled FA-PLP-^131^I and PLP-^131^I nanoparticles (Additional file 1: Figure S1). These results demonstrate the high cellular uptake of FA-PLP-^131^I, likely due to the FA-mediated targeting effect on B16F10 cells.

### In Vitro Cytotoxicity

**Fig. 5:**
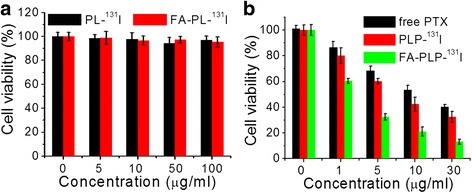
**a** Cell viability of B16F10 cells after treatment with PL-^131^I and FA-PL-^131^I for 24 h. **b** Cell viabilities of B16F10 cells after incubation with different concentrations of free PTX, PLP-^131^I, and FA-PLP-^131^I for 24 h

The cytotoxicity of the control nanocarriers, PL-^131^I, and FA-PL-^131^I was tested by MTT assay. Cells treated with PL-^131^I and FA-PL-^131^I for 24 h had a similar viability to the control (Fig. 5a), indicating a good biocompatibility. Additionally, FA-PLP-^131^I was much more effective at suppressing B16F10 cell proliferation than free PTX and PLP-^131^I at the same concentration of PTX (Fig. 5b), indicating excellent cell-targeted chemotherapy. The results demonstrate that FA-PLP-^131^I has a high chemotherapeutic effect with no radiotoxicity and cytotoxicity.

### Blood Circulation and Biodistribution Study

**Fig. 6:**
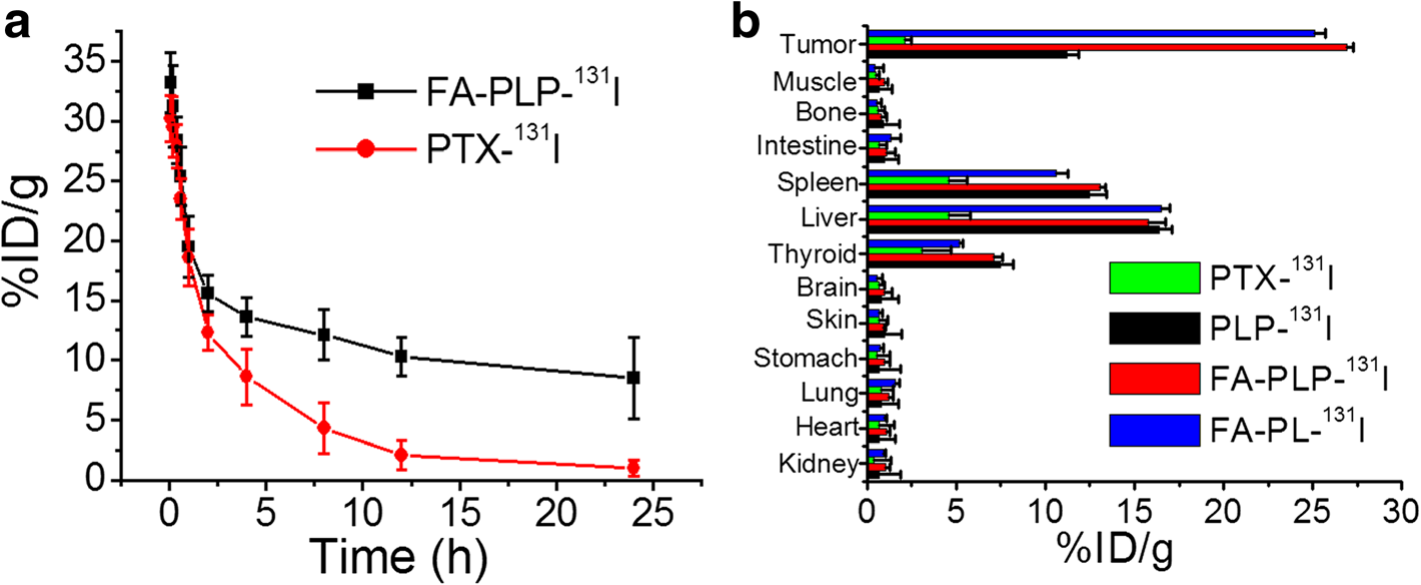
**a** The blood circulation curve of FA-PLP-^131^I after intravenous injection. **b** Biodistribution of PTX-^131^I, FA-PL-^131^I, FA-PLP-^131^I, and PLP-^131^I in B16F10 tumor-bearing mice

Radiolabeling has been reported to be more reliable than fluorescence imaging for the quantitative and accurate in vivo tracking of nanoparticles [27, 28]. ^131^I-labeled FA-PLP nanoparticles were prepared to investigate their in vivo behavior, including blood circulation and biodistribution, as measured by a gamma counter. The blood circulation half-life of free PTX (*t*_1/2_ = 5.4 ± 0.23 h) was prolonged to 18.5 ± 0.5 h by FA-PLP-^131^I (Fig. 6a) due to nanoparticle encapsulation, which was favorable for tumor targeting accumulation [29–31]. Next, the biodistribution of free PTX-^131^I, PLP-^131^I, and FA-PLP-^131^I in B16F10 tumor-bearing mice at 1 day post injection was investigated (Fig. 6b). FA-PLP-^131^I, as well as FA-PL-^131^I, exhibited an obvious enhancement of tumor uptake, which was 4.41- and 12.8-fold than that of PLP-^131^I and free PTX-^131^I, respectively, likely due to the prolonged blood circulation of FA-PLP-^131^I, FA-PL-^131^I, and its FA targeting effect. In addition, the liver and spleen also exhibited a relatively high uptake due to their nanoparticle metabolism, which were the normal metabolic organs [32, 33].

### In Vivo Tumor Chemotherapy

**Fig. 7:**
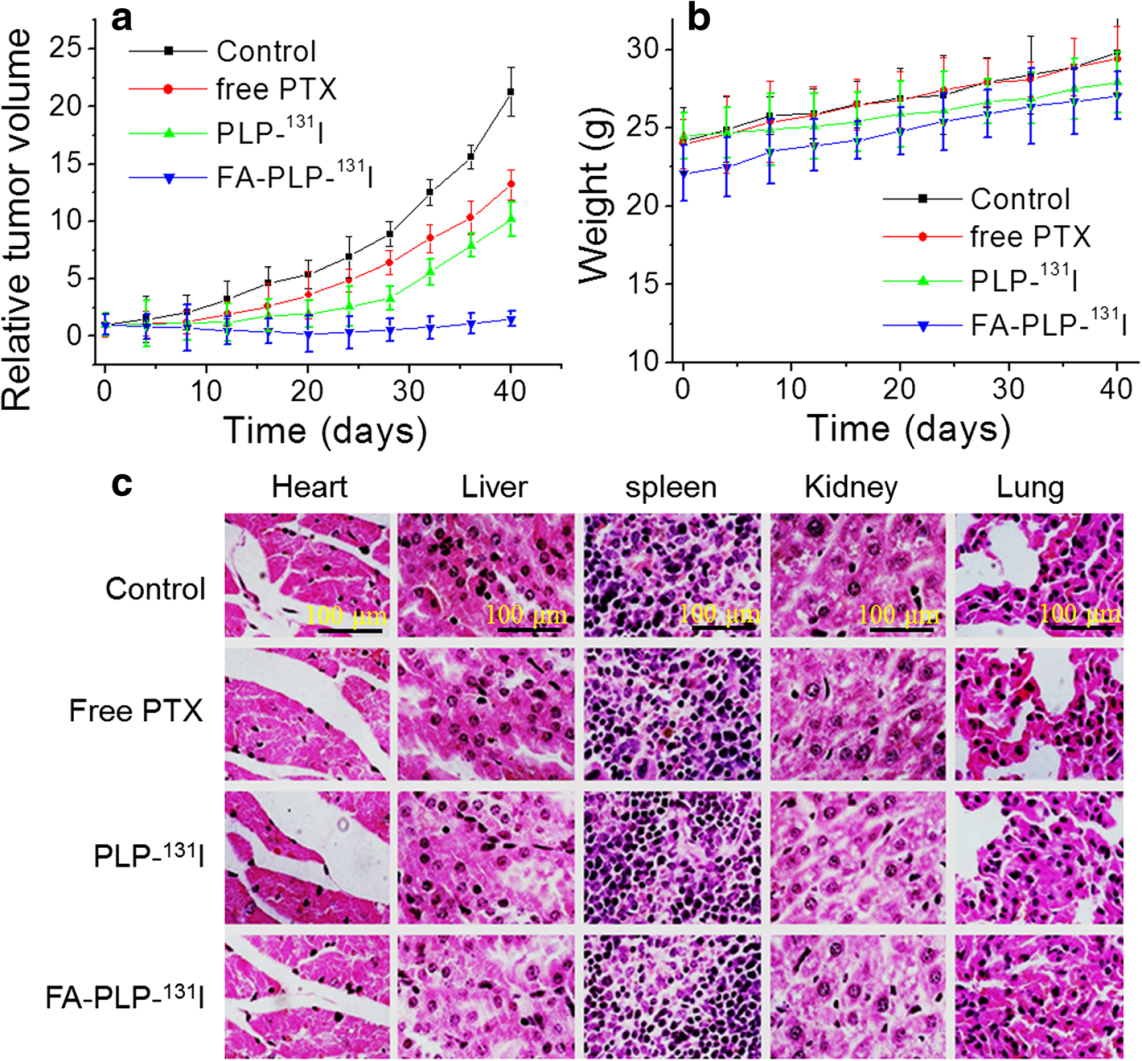
**a** The relative tumor volume and **b** body weight of tumor-bearing mice after tail vein injection with saline (control), free PTX, PLP-^131^I, and FA- PLP-^131^I. **b** Body weight of tumor-bearing mice after tail vein injection with saline (control), free PTX, PLP-^131^I, and FA- PLP-^131^I. **c** The representative hematoxylin and eosin staining images of major organs including the heart, liver, spleen, lung, and kidney. *Scale bar* = 100 μm

Free PTX-, PLP-^131^I-, and FA-PLP-^131^I-treated mice exhibited inhibition of tumor growth as compared to the saline control group (Fig. 7a). Overall, FA-PLP-^131^I was the most effective at tumor growth suppression without relapse compared to all treated groups after approximately 40 days of treatment. These results are as expected considering the significantly prolonged blood circulation of FA-PLP-^131^I and therefore its ability to promote FA-mediated tumor targeted accumulation. Further, tumor-targeted accumulation also led to a reduction in peripheral exposure of PTX, thereby minimizing systemic toxicity. As expected, over the complete treatment process, there was no noticeable loss in body weight (Fig. 7b), and the major organs, including the heart, liver, spleen, lung, and kidney, showed no obvious histological lesions in any group (Fig. 7c).

## Conclusions

In summary, we have synthesized ^131^I-labeled PLGA-lipid nanoparticles as drug delivery carriers for melanoma-targeted chemotherapy. By measuring the radioactivity of ^131^I, the in vitro and in vivo behaviors of FA-PLP-^131^I nanoparticles were studied. The obtained FA-PLP-^131^I showed great dispersity and colloidal and radiolabeling stability. The control nanocarriers, PL-^131^I, and FA-PL-^131^I also showed good biocompatibility. Following encapsulation of PTX, FA-PLP-^131^I was the most effective at suppressing B16F10 cell proliferation without cytotoxicity, attributed to the FA targeting effect. Moreover, FA-PLP-^131^I was demonstrated to significantly prolong the blood circulation of PTX and to effectively accumulate within the targeted tumor region. Thus, FA-PLP-^131^I had an excellent tumor growth inhibition and great in vivo biocompatibility. The results highlight the promising potential of these versatile FA-PLP-^131^I nanocarriers as reliable drug tracking agents as well as their application in tumor targeted therapy.

## Additional file

Additional file 1: Cell uptake data obtained by confocal laser scanning microscopy. (DOCX 150 kb)
