# Supplementary material for: 131I-Traced PLGA-Lipid Nanoparticles as Drug Delivery Carriers for the Targeted Chemotherapeutic Treatment of Melanoma
Source: Nanoscale Res Lett. 2017 May 19;12:365. doi: 10.1186/s11671-017-2140-7 (PMC5438325; doi:10.1186/s11671-017-2140-7)
Supplement: Additional file 1: — Cell uptake data obtained by confocal laser scanning microscopy. (DOCX 150 kb) [file 11671_2017_2140_MOESM1_ESM.docx]

**Supplementary Material**

**^131^I-traced PLGA-lipid nanoparticles as drug delivery carriers for the targeted chemotherapeutic treatment of melanoma**

**Haiyan Wang^1^ and Weizhong Sheng^2^***

^1^ Department of Nuclear Medicine, Shanghai East Hospital, Tongji University School of Medicine, Shanghai 200120, China

^2^ Department of General Surgery, Zhongshan Hospital, Fudan University, Shanghai 200032, China

Corresponding author: Weizhong Sheng, Email: shengwz_gs@yeah.net

**Tab. S1** The radiolabeled yield of various samples.

|  | PTX-^131^I | PL-^131^I | PLP-^131^I | FA-PL-^131^I | FA-PLP-^131^I |
| --- | --- | --- | --- | --- | --- |
| Radiolabeled yield (%) | 45.6±2.3 | 52.1±4.1 | 48.9±1.9 | 56.3±2.5 | 54.8±2.7 |

*
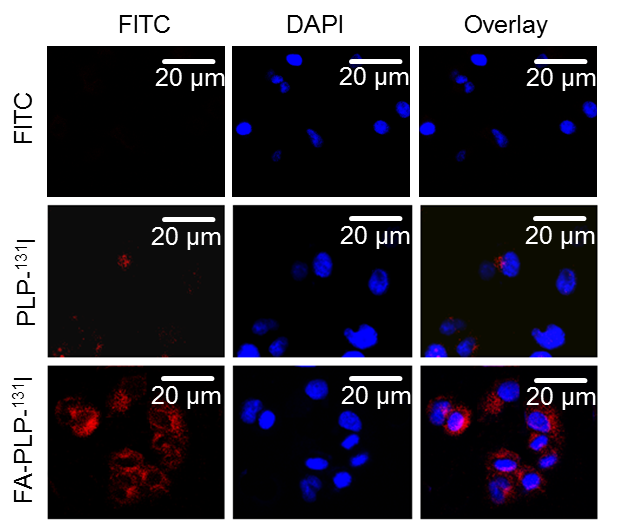
*

**Fig. S1** Confocal laser scanning microscopy images of B16F10 cells incubated with free FITC, FITC-labeled FA-PLP-^131^I and PLP-^131^I nanoparticles.
